# An Anti-Interference Scheme for UAV Data Links in Air–Ground Integrated Vehicular Networks

**DOI:** 10.3390/s19214742

**Published:** 2019-10-31

**Authors:** Yixin He, Daosen Zhai, Ruonan Zhang, Xiaojiang Du, Mohsen Guizani

**Affiliations:** 1 Department of Communication Engineering, Northwestern Polytechnical University, Xi’an 710072, China; jhlhhyx@mail.nwpu.edu.cn (Y.H.); rzhang@nwpu.edu.cn (R.Z.); 2 Department of Computer and Information Sciences, Temple University, Philadelphia, PA 19122, USA; dxj@ieee.org; 3 Department of Computer Science and Engineering, Qatar University, Doha 2713, Qatar; mguizani@ieee.org

**Keywords:** air–ground integrated vehicular networks (AGIVNs), data links, multi-ary (M-ary) spread spectrum, multi-carrier modulation (MCM), unmanned aerial vehicle (UAV)

## Abstract

As one of the main applications of the Internet of things (IoT), the vehicular ad-hoc network (VANET) is the core of the intelligent transportation system (ITS). Air–ground integrated vehicular networks (AGIVNs) assisted by unmanned aerial vehicles (UAVs) have the advantages of wide coverage and flexible configuration, which outperform the ground-based VANET in terms of communication quality. However, the complex electromagnetic interference (EMI) severely degrades the communication performance of UAV sensors. Therefore, it is meaningful and challenging to design an efficient anti-interference scheme for UAV data links in AGIVNs. In this paper, we propose an anti-interference scheme, named as Mary-MCM, for UAV data links in AGIVNs based on multi-ary (M-ary) spread spectrum and multi-carrier modulation (MCM). Specifically, the Mary-MCM disperses the interference power by expanding the signal spectrum, such that the anti-interference ability of AGIVNs is enhanced. Besides, by using MCM and multiple-input multiple-output (MIMO) technologies, the Mary-MCM improves the spectrum utilization effectively while ensuring system performance. The simulation results verify that the Mary-MCM achieves excellent anti-interference performance under different EMI combinations.

## 1. Introduction

In recent years, with the rapid development of the Internet of things (IoT) industry and the rise of some new businesses such as smart society, smart parks and smart cities, the world has entered the era of the Internet of everything [1]. Specifically, IoT has penetrated every aspect of life, including medicine, environment, agriculture, transportation, and education [2].

The vehicular ad-hoc network (VANET) is one of the main applications of the IoT, which has received extensive attention as the core of the intelligent transportation systems (ITS) [3]. VANET can provide users with various types of services through sensors, including road safety, entertainment, and path planning [4]. In ITS, the vehicle not only needs to acquire a wide range of traffic conditions and warning information in real time, but also needs to transmit this information. However, in larger spatial scales, obstacles, electromagnetic interference, and bad weather may lead to the quality decline or even breakdown of the data link [5]. In some extreme environments, due to the lack of infrastructure, it is sometimes difficult to meet the demand only based on the ground-based VANET [6,7]. In the future, ITS applications will be supported by a new VANET architecture with greater coverage and more flexible, efficient means of communication.

Inspired by the above, we propose an air–ground integrated vehicular networks (AGIVNs) architecture that utilizes UAV-assisted communication to improve the quality of VANET communication. AGIVNs are typically composed of many low-cost and low-power sensors, which can perform sensing, simple computations, and short-range wireless communications [8]. Sensors mounted on UAVs or vehicles can intelligently detect road conditions, such as real-time traffic congestion, average speed, surface condition, or high-speed tolling. The information obtained through sensors can be forwarded to the driver via AGIVNs to assist the driver to avoid collisions at crowded intersections and highway entries [9]. From the above, the information acquisition, processing, and transmission are three important functions of the sensors in AGIVNs.

However, with the rapid growth of wireless services and mobile devices, electromagnetic interference (EMI) is increasingly serious [10]. At present, the main anti-interference technologies such as direct sequence spread spectrum (DSSS), frequency-hopping spread spectrum (FHSS), and time hopping (TH) cannot solve the EMI problem of AGIVNs [11]. Therefore, for the sensor information transmission, developing radio access technologies (RATs) that enable reliable and low-latency AGIVNs communications has become a hot topic.

In recent years, some researchers have proposed that vehicle-to-everything (V2X) communications can improve the reliability of communication services, lower the end-to-end latency and support applications that require high throughput. For instance, the authors of [12] indicated that V2X communications have the potential to significantly bring down the number of vehicle crashes, thereby reducing the number of associated fatalities. In addition, V2X-capable vehicles can assist in better traffic management leading to greener vehicles and lower fuel costs [13,14]. Most of them have been devoted to improving the performance of transmission technologies for V2X. However, AGIVNs are different from VANET, the transmission technologies for V2X are difficult to guarantee the accurate transmission of information in the environment of strong EMI. Because the transmission technologies for V2X do not take account of the impacts of UAV data links on AGIVNs performance.

In this paper, different from aforementioned schemes, we take into consideration the characteristics of spreading and utilize multi-ary (M-ary) spread spectrum to improve the spectrum utilization of the UAV data link. M-ary spread spectrum can use orthogonal variable spreading factor (OVSF) to transmit information. At the same baud rate and spreading gain, the system bandwidth of M-ary is only $1/\log_{2}M$ of DSSS. In addition, we also adopt the multi-carrier modulation (MCM) technology to improve the anti-interference performance. Specifically, MCM splits the data stream into several substreams, thus dispersing the interference signal. Therefore, the anti-interference scheme combining M-ary spread spectrum and MCM can not only obtain the same anti-interference performance as DSSS, but also improve spectrum utilization effectively.

Motivated by the above reasons, we propose an anti-interference scheme for the UAV data links in AGIVNs based on M-ary spread spectrum and MCM, named as Mary-MCM. In this paper, we analyze the EMI of UAV data link, establish the EMI model, and classify different EMI types. Then, we apply the Mary-MCM anti-interference scheme into the sensor transmitter (STX) and the sensor receiver (SRX). In this respect, Mary-MCM scheme enables efficient message delivery and effectively limits the symbol error rate (SER). Furthermore, we performed extensive simulations to evaluate the performance of our proposed anti-interference scheme under unusual interference combinations.

Compared with the existing anti-interference technology, the proposed Mary-MCM scheme has the following advantages

- We adopt M-ary spread spectrum technology to expand the spectrum of the signal and disperse the interference power of the signal. Therefore, the accuracy of information transmission can be improved and the bit error rate (BER) can be reduced without increasing the transmission power.
- We adopt multi-carrier technology to modulate the signal and send it through multiple-input multiple-output (MIMO) antennas. Therefore, Mary-MCM scheme can improve channel capacity and spectrum utilization. In addition, Mary-MCM scheme can be used in IoT environments with limited radio spectrum resources to meet the user’s demand for multiple services and large capacity.
- Compared with transmission technologies for V2X, Mary-MCM scheme aims to improve anti-interference performance of UAV data links. Considering the influence of EMI in three-dimensional (3D) environment, the proposed Mary-MCM scheme can improve the reliability of communication services, lower the end-to-end latency, and support applications that require high throughput.

The rest of the paper is organized as follows. Section 2 introduces the principle of anti-interference technology and summarizes the advantages and disadvantages of the existing technology. In Section 3, we model and analyze the EMI in AGIVNs. Section 4 proposes the Mary-MCM scheme and analyzes the anti-interference performance of Mary-MCM scheme theoretically. Extensive simulations are present in Section 5 to measure the performance of Mary-MCM by comparing it with DSSS. Finally, the conclusions and future works are presented in Section 6.

## 2. Related Technologies and Works

In this section, we summarize the principles of anti-interference technologies of the UAV data link and introduce the commonly used anti-interference technologies.

### 2.1. Anti-Interference Technology Principle

Anti-interference communication means that effective information transmission can still be carried out under various man-made or natural EMI. In AGIVNs, communication signals from vehicles or UAVs overlap with interference signals in time domain, frequency domain and power domain. When the interference signal and communication signals overlap, it is necessary to use anti-interference technology to eliminate the interference, the purpose is to increase the signal-to-interference ratio (SIR) of the SRX. According to the Shannon formula, the channel capacity of the SRX is

(1) $$C=B\log_{2}\left(1+\frac{S}{N}\right),$$

where *N* is the noise power (W), *C* is the channel capacity (bit/s), *S* is the signal power (W), and *B* is the channel bandwidth (Hz).

When N/S≥1, we can get

(2) $$B\approx 0.7C\frac{N}{S}.$$

As can be seen from Equation (2), when the noise power and the signal power ratio (N/S) are fixed, the channel capacity increases linearly with the bandwidth. In other words, improving the system bandwidth can improve the anti-interference performance of the system. Currently commonly used anti-interference technologies mainly include the DSSS technology and the M-ary spread spectrum technology [15].

### 2.2. DSSS Technology

**Figure 1:**
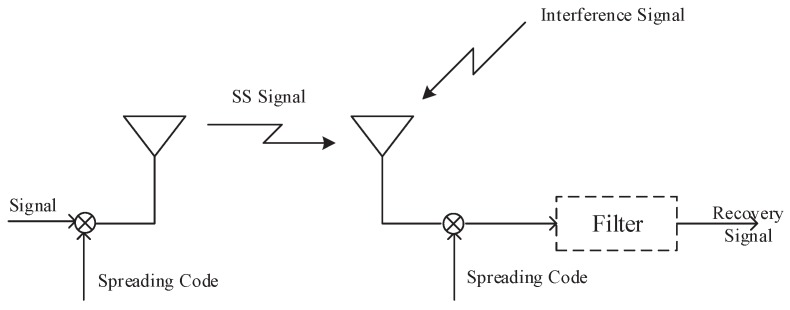
DSSS technology schematic.

The purpose of DSSS is to expand the signal bandwidth. On the premise of ensuring synchronization between the STX and the SRX, SRX uses the same pseudo code sequence in exclusive OR (XOR) processing to obtain the transmitted information. Figure 1 is the schematic diagram of DSSS technology.

The STX signal is x(t) and the spread spectrum signal is g(t); the signal after spreading is

(3) f(t)=x(t)g(t).

We take the Fourier transform (FT) into the frequency domain.

(4) F(ω)=X(ω)G(ω).

Then, we adopt binary phase shift keying (BPSK) modulation for the signal; the transmitted signal s(t) can be expressed as

(5) $$s\left(t\right)=\sqrt{2P}x\left(t\right)g\left(t\right)\cos\omega_{0}t,$$

where *P* is the carrier power and $\omega_{0}$ is the angular frequency.

Finally, we unpack and filter the information on SRX, which can be expressed as

(6) $$s^{\prime }\left(t\right)=A\sqrt{2P}x\left(t-T_{d}\right)g\left(t-T_{d}\right)g\left(t-T_{d}^{\prime }\right)\left[\cos\omega_{0}\left(t-T_{d}\right)+\varphi \right],$$

where $T_{d}$ is the information delay, *A* is the spreading gain, φ is the random phase, $g(t-T_{d}^{\prime })$ is the despreading sequence, and $T_{d}^{\prime }$ is the information delay estimate, when $T_{d}=T_{d}^{\prime }$, $g\left(t-T_{d}\right)g\left(t-T_{d}^{\prime }\right)=1$.

The authors of [16] proposed a communication system based on the DSSS to reduce the BER by increasing STX power. L. Xiao et al. [17] used chaotic map sequences to improve the security of the DSSS anti-interference ability of the system without increasing the spread spectrum sequence. In addition, the DSSS is applicable to the actual engineering field by using field-programmable gate array (FPGA) [18].

Although the DSSS technology is easy to implement, it has some disadvantages. Because DSSS bandwidth is large, it is vulnerable to EMI. For instance, when the intensity of interference signal exceeds the gain range of DSSS, the system will not transmit the information correctly [19].

### 2.3. M-Ary Spread Spectrum

**Figure 2:**
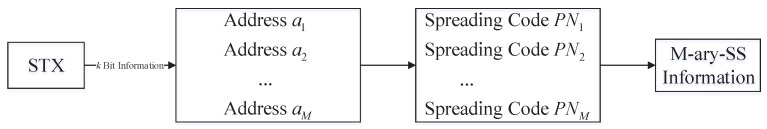
M-ary spread spectrum technology schematic.

The DSSS system improves the anti-interference performance of the system by improving the transmission bandwidth of the system. However, in a real scenario, the system bandwidth is limited and cannot be expanded arbitrarily. Therefore, we use the M-ary spread spectrum to increase the spreading gain. Figure 2 is a schematic diagram of the M-ary spread spectrum technology [20].

As shown in Figure 2, the STX needs to transmit *k* bits information, and convert the *k* bits information into *M* addresses ($M=2^{k}$). Each address corresponds to a pseudo-noise (PN) code, and PN codes are orthogonal to each other.

DSSS spreading gain can be expressed as

(7) $$G_{p}\left(dB\right)=10\log_{10}\left(N_{p}\right),$$

where $N_{p}$ is the spreading code length of DSSS.

The PN codes of the M-ary spread spectrum correspond to the *k* bits information, thus the gain of the M-ary spread spectrum can be expressed as

(8) $$G_{d}\left(dB\right)=G_{s}+G_{c},$$

where $G_{c}$ is the coding gain and $G_{s}$ is the spreading gain. $G_{s}$ can be expressed as

(9) $$G_{s}\left(dB\right)=10\log_{10}\left(\frac{N_{d}}{k}\right),$$

where $N_{d}$ is the spread code length of M-ary spread spectrum.

(10) $$G_{c}\left(dB\right)=10\log_{10}\left(k\right).$$

Compared with DSSS, M-ary spread spectrum has not only spreading gain but also specific coding gain. Therefore, M-ary spread spectrum can improve the anti-interference performance of the UAV data link. The authors of [21] indicated that the use of M-ary spread spectrum can effectively improve the channel capacity, and proposed a hybrid M-ary orthogonal spread spectrum technology based on M-ary spread spectrum and differential modulation. M-ary spread spectrum can improve the transmission performance of the system at the same signal bandwidth and transmission rate. Ding et al. [22] realized this scheme through FPGA. In addition, Mossallamy analyzed the error performance of the M-ary spread spectrum system [23], and proved that M-ary spread spectrum has good performance in the communication system with limited bandwidth.

### 2.4. Summary

Many studies have been made on the spread spectrum system, including the combination of DSSS and FHSS to form DSSS/FHSS technology. However, using FHSS technology can reduce the security of the system. Therefore, it is difficult to ensure the security requirements of AGIVNs by using DSSS/FHSS anti-interference scheme. Compared to the DSSS/FHSS anti-interference scheme, Mary-MCM has higher spectrum efficiency and security. On the one hand, the baud rate of Mary-MCM is only $1/\log_{2}M$ of DSSS, which reduces the system speed requirement. On the other hand, from the spectrum of the signal, the spectrum of the Mary-MCM signal is closer to white noise, which enhances the security of the system.

In addition, we find that most anti-interference schemes are based on the research of spread spectrum code. At present, no scholars have put forward an anti-interference scheme that combines multi-base spread spectrum with multi-carrier modulation. Based on the predecessors, this paper proposes an UAV data link anti-interference scheme based on M-ary spread spectrum and MCM.

## 3. Air–Ground Integrated Vehicular Networks (AGIVNs) UAV Data Links

In this section, we model and analyze the EMI in AGIVNs, and we also simulate the common EMI.

### 3.1. UAV Data Links Electromagnetic Interference Analysis

AGIVNs use UAVs to assist vehicles communication. The UAV data link is the key link between the UAV’s information transmission and the vehicle, the UAV and the base station during the mission. It bears the significant task of UAV control command and information transmission. The performance of the UAV data link directly influences the performance of AGIVNs.

**Figure 3:**
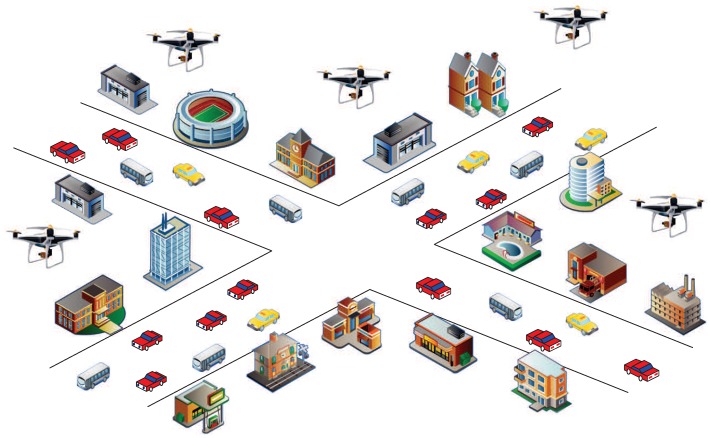
Air–ground integrated vehicular networks.

Figure 3 is the schematic diagram of AGIVNs. In the real world, hundreds of heterogeneous IoT sensors will be deployed in the region. These sensors perform tasks through data links in various applications.

**Figure 4:**
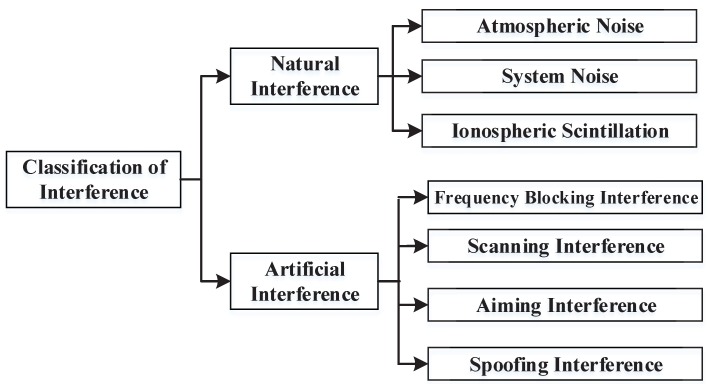
Electromagnetic interference classification.

However, when the UAV communicates, it often faces various EMI [24], such as co-channel interference caused by communication equipment, atmospheric noise interference in the natural environment, or human interference caused by human activities. At present, there are numerous classification methods for EMI. This paper classifies type interference according to Figure 4.

### 3.2. Electromagnetic Interference Model

Through the analysis of the previous section, we can know that the signal received at the STX is composed of two parts, the transmitted signal and the interference signal, which can be expressed as

(11) y(t)=s(t)+n(t),

where s(t) is the transmitted signal and n(t) is the interference signal.

This paper models common interfering signals and analyzes their impact on the UAV data link.

#### 3.2.1. Radar Pulse (RP) Interference

**Figure 5:**
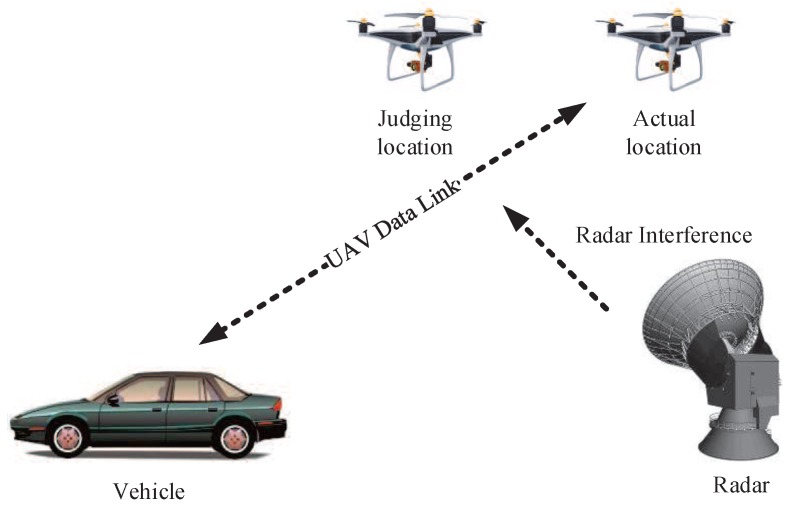
RP interference.

Radar pulse (RP) interference is mainly caused by terrestrial radar transmitting wireless electromagnetic waves. Due to RP interference, ground vehicles are unable to accurately obtain the location of the UAV. Figure 5 is the schematic diagram of RP interference.

RP interference can be expressed as

(12) $$\left.\begin{array}{c} s\left(t\right)=u_{i}\left(t\right)e^{j2\pi f_{0}t}\\ u_{i}\left(t\right)=A\cdot rect\left(t/\tau \right) \end{array}\right\},$$

where *A* is the pulse signal amplitude, $f_{0}$ is the signal frequency, rect(·) is the matrix pulse function, and τ is the pulse width.

**Figure 6:**
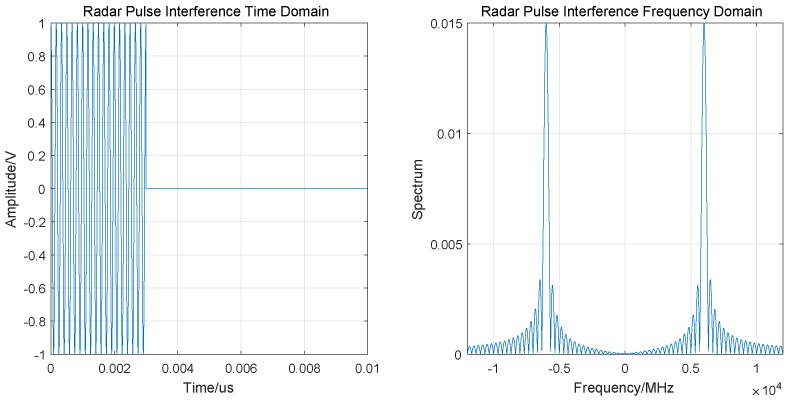
RP interference time–frequency domain simulation.

We simulated it in the time–frequency domain and the results are shown in Figure 6.

#### 3.2.2. Communication Radiation Source (CRS) Interference

**Figure 7:**
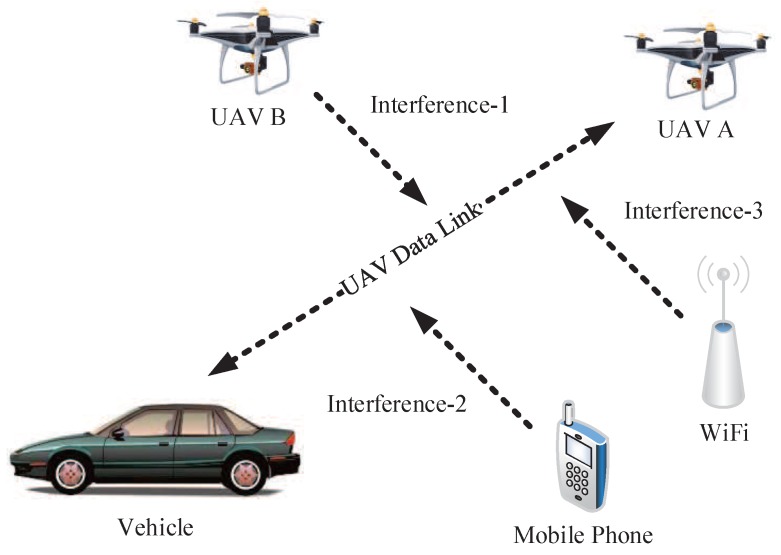
CRS interference.

UAV data links can be disrupted by communication radiation source (CRS) interference from mobile phones and Wi-Fi. CRS interference affects the received signal and increases the BER. Figure 7 is the schematic illustration of CRS interference.

CRS interference can interfere with the amplitude, frequency and phase of the signal. Amplitude interference is taken as an example, which can be expressed as

(13) $$S_{AM}\left(t\right)=\left[A_{0}+f\left(t\right)\right]\cos\left(2\pi f_{c}t+\theta_{c}\right),$$

where $A_{0}$ is the DC component, f(t) is the modulated signal, $f_{c}$ is the carrier frequency, and $\theta_{c}$ is the initial phase.

**Figure 8:**
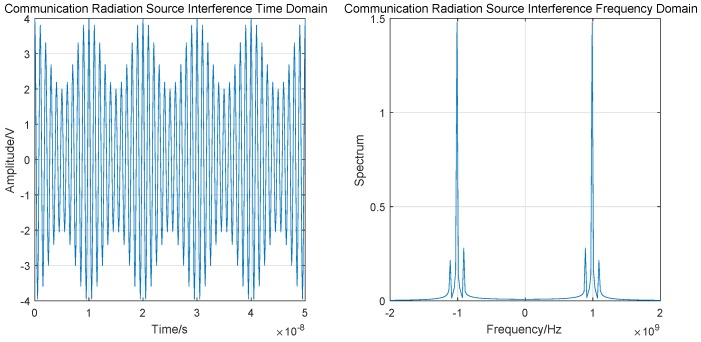
CRS interference time–frequency domain simulation.

The time–frequency domain simulation results are shown in Figure 8.

#### 3.2.3. Single Frequency Continuous Wave (SFCW) Interference

**Figure 9:**
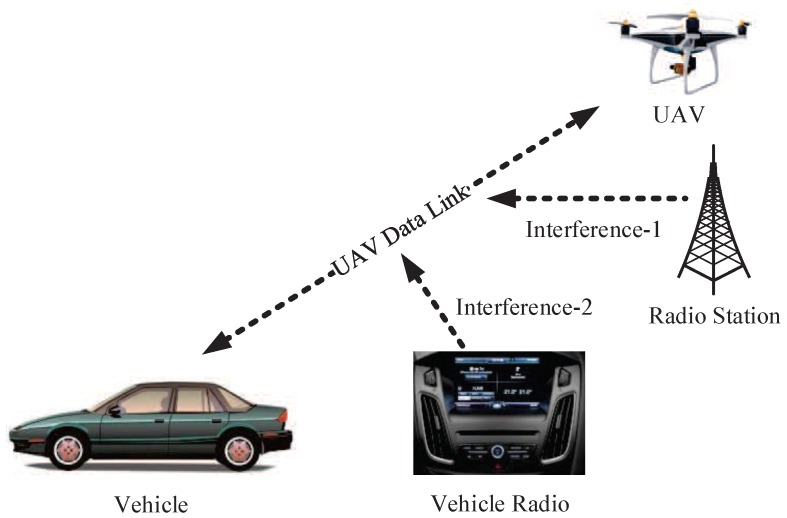
SFCW interference.

In AGIVNs radio stations, vehicle radios often generate single frequency continuous wave (SFCW). Thus, when this signal frequency is equal to the center frequency of the UAV data link, it affects the reception of the signal. Figure 9 is the schematic illustration of SFCW interference.

SFCW can be expressed as

(14) $$S\left(t\right)=A\exp\left[j2\pi f_{0}t+\varphi \right],$$

**Figure 10:**
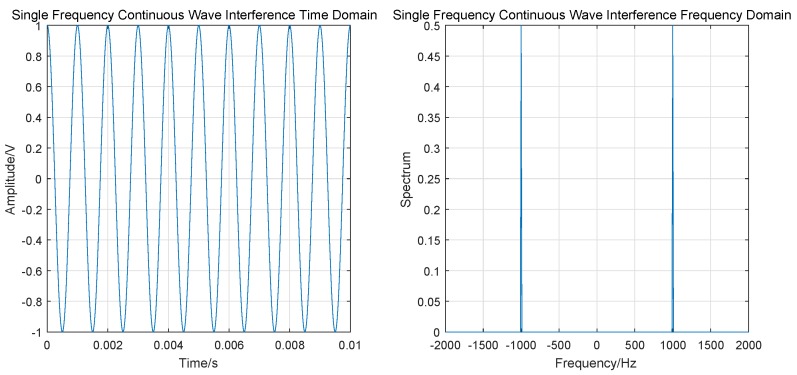
SFCW interference time–frequency domain simulation.

The time–frequency domain simulation results are shown in Figure 10.

## 4. Mary-MCM UAV Data Links Anti-Interference Scheme

**Figure 11:**
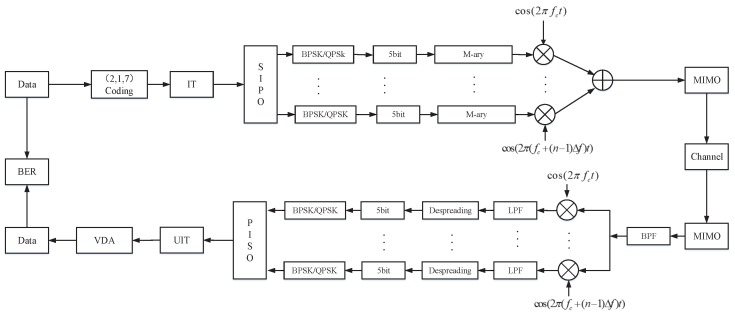
Mary-MCM scheme overall flow chart.

In this section, we describe the Mary-MCM scheme, which mainly includes three parts: the STX system, the SRX system, and the sensor MIMO antenna system. The overall scheme flow chart is shown in Figure 11. In addition, we also analyze the anti-interference performance of Mary-MCM scheme.

### 4.1. Sensor Transmitter (STX) System

**Figure 12:**
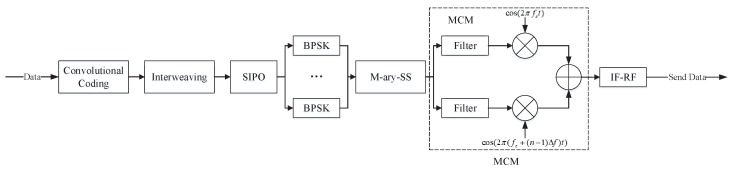
STX system block diagram.

Figure 12 is the block diagram of the STX system of the Mary-MCM scheme. The STX has *k* parallel transmission link ports, and can realize real time input and output for data separately. Furthermore, STX adopts BPSK modulation for data. Moreover, STX uses MCM technology to process the data.

**Figure 13:**
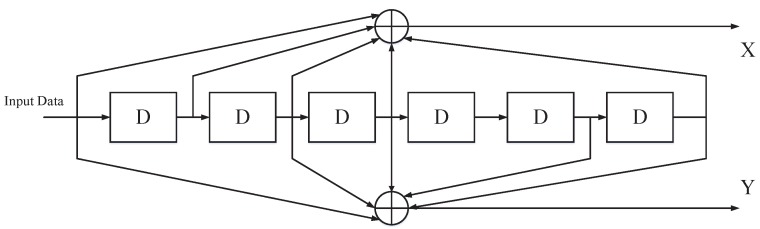
Convolutional coding module block diagram.

Specifically, the data rate input of STX is $1/T_{b}$. Then, STX uses the (2, 1, 7) convolutional coding to encode the data. Figure 13 is the convolutional coding module block diagram.

After passing through the convolutional coding module, the data rate becomes $2/T_{b}$. STX transforms high bit-rate (a(t)) into low symbol-rate ($a_{k}\left(t\right)$) with the serial input parallel output (SIPO) module. At this point, the data rate is $1/(2\times T_{b})$. The relationship between a(t) and $a_{k}\left(t\right)$ can be expressed as

(15) $$a\left(t\right)=\sum_{i=-\infty }^{+\infty }a_{k}\left(t-iT\right).$$

**Figure 14:**
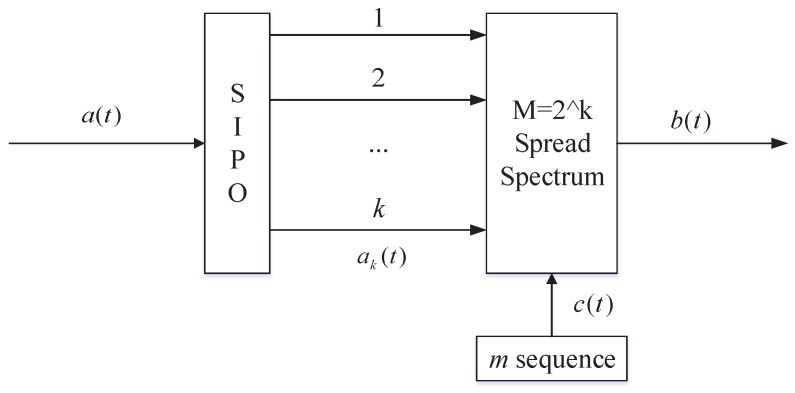
M-ary spread spectrum module block diagram.

The low symbol-rate data are processed by MCM after BPSK modulation. Figure 14 is the M-ary spread spectrum module block diagram.

The $a_{k}\left(t\right)$ can generate spread spectrum code b(t) after passing through the M-ary spread spectrum module. We adopt *m* sequence (c(t)) as spread spectrum sequence. The *m* sequence is a pseudo-random sequence with good autocorrelation and orthogonal mutual correlation. The generator polynomial of *m* sequence can be expressed as

(16) $$f\left(x\right)=x^{7}+x+1.$$

The length of *m* sequence is 128, which is easy to implement and can effectively reduce the complexity of STX. In addition, the pseudo-randomness of *m* sequence can improve the anti-interference ability of STX. Therefore, the generated spreading code can be expressed as

(17) $$b\left(t\right)=\sum_{i=0}^{\infty }c_{i}\left(t-iT\right).$$

Finally, the signal after MCM modulation can be expressed as

(18) $$s\left(t\right)=\sum_{i=1}^{M}\sqrt{2P_{s}}c_{i}\left(t\right)e^{jw_{i}t},$$

where $P_{s}$ is the UAV data link transmit power.

### 4.2. Sensor Receiver (SRX) System

**Figure 15:**
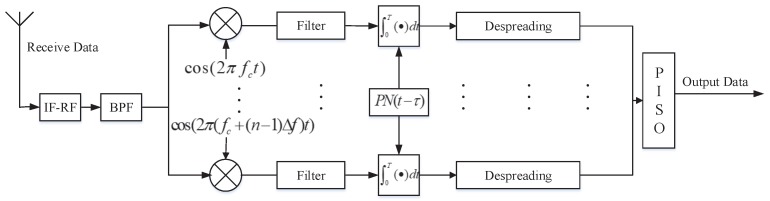
SRX system block diagram.

Figure 15 is the block diagram of the SRX system of the Mary-MCM scheme.

After the transmitting signal passing through the wireless channel, the signal reaching SRX can be expressed as

(19) r(t)=s(t−τ)+n(t),

where τ is the transmission delay and n(t) is the interference signal.

In Mary-MCM scheme, r(t) first needs to filter clutter and then multiply with subcarriers to restore the signal. The restoring signal $d_{k}\left(t\right)$ can be expressed as

(20) $$d_{k}\left(t\right)=r\left(t\right)e^{-j2\pi f_{k}t}.$$

Then, we despread the restoring signal at SRX, and the restoring signal after despreading $D_{k}\left(t\right)$ can be expressed as

(21) $$D_{k}\left(t\right)=\int_{0}^{T}c\left(t\right)d_{k}\left(t\right)dt.$$

Finally, the receiver can complete acceptance by passing $D_{k}\left(t\right)$ through the parallel input serial output (PISO) module.

### 4.3. Sensor Multiple-Input Multiple-Output (MIMO) Antenna System

In AGIVNs, sensor nodes (UAVs or vehicles) need to exchange information with multiple neighboring sensor nodes at the same time. Therefore, single-input single-output (SISO) technology is difficult to meet the needs of data transmission. For the above reasons, we use MIMO technology to increase channel capacity.

Assume that the STX system uses *T* antennas, and the SRX system uses *R* antennas. The signal that we receive in SRX is

(22) $$r_{M}\left(t\right)=H_{T*R}s\left(t-\tau \right)+n\left(t\right),$$

where $H_{T*R}$ is the *T*∗*R* dimensional channel matrix, which can be expressed as

(23) $$H_{T*R}=\left[\begin{array}{cccc} h_{11} & h_{12} & \ldots & h_{1R}\\ h_{21} & h_{22} & \ldots & h_{2R}\\ \ldots & \ldots & \ldots & \ldots \\ h_{T1} & h_{T2} & \ldots & h_{TR} \end{array}\right],$$

where $h_{TR}$ is the spatial channel gain.

**Figure 16:**
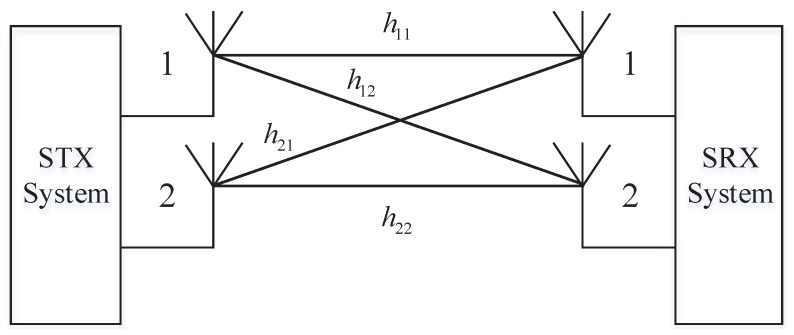
Dual-polarized 2 × 2 MIMO antenna system.

Mary-MCM scheme uses 2 × 2 MIMO technology to realize communication, and two circularly polarized antennas, respectively, generate left/right circularly polarized waves. As shown in Figure 16, two antennas are used in STX: the left-handed circularly polarized antenna and the right-handed circularly polarized antenna. It corresponds to that two left/right circularly polarized antennas identical to the STX are installed at the SRX to form a dual-polarized 2 × 2 MIMO antenna systems.

The channel matrix of the dual-polarized 2 × 2 MIMO antenna system can be expressed as

(24) $$H_{2*2}=\left[\begin{array}{cc} h_{11} & h_{12}\\ h_{21} & h_{22} \end{array}\right].$$

For the 2 × 2 MIMO antenna system, the channel capacity can be expressed as

(25) $$C_{MIMO}=\mathrm{lo}g_{2}\det\left[I_{2*2}+\frac{SNR}{n_{T}}H_{2*2}H_{2*2}^{H}\right],$$

where $I_{2*2}$ is the 2×2 dimensional unit matrix and $H_{2*2}^{H}$ is the conjugate transposed matrix of $H_{2*2}$.

### 4.4. Anti-Interference Performance Analysis

We take white Gaussian noise (WGN) as an example to analyze the anti-interference performance of Mary-MCM scheme. The power spectral density of WGN is $N_{0}/2$ and the mean value is zero. Therefore, the restoring signal disturbed by WGN can be expressed as

(26) $$d_{k-W}\left(t\right)=\left[c_{i}\left(t\right)+n\left(t\right)+s\left(t\right)\right]e^{j\varphi_{0}},$$

where $\varphi_{0}$ is the phase deviation caused by WGN.

The dispreading signal is

(27) $$D_{k-W}\left(t\right)=e^{j\varphi_{0}}\int_{0}^{T}[c_{k}\left(t\right)+n\left(t\right)+s\left(t\right)]c_{k}\left(t\right)dt=e^{j\varphi_{0}}\eta_{k}\left(t\right).$$

It can eliminate the effects of phase deviation by modulo operation (MO), thus Equation (27) can be expressed as

(28) $$V_{k}\left(t\right)=|D_{k-W}\left(t\right)|=|\eta_{k}\left(t\right)|.$$

It can be known from the pseudo-randomness of *m* sequence that, when $d_{k-W}\left(t\right)=d_{k}\left(t\right)$, the value of $V_{k}\left(t\right)$ is the largest. In addition, according to the central limit theorem, $\eta_{k}\left(t\right)$ obeys Gaussian distribution. Therefore, the SER $P_{M}$ can be expressed as

(29) $$P_{M}=\left(M-1\right)Q\left[{\left(\frac{N_{0}}{E_{s}}+\frac{2(k-1)}{3NT_{c}^{3}}\right)}^{-1/2}\right],$$

where $E_{s}$ is the symbol energy, $N_{0}$ is the WGN power spectral density, $T_{c}$ is the chip interval, $Q\left(x\right)\;=\;\frac{1}{\sqrt{2}\pi }\int_{x}^{\infty }e^{-1/2t^{2}}dt$, and $k=\log_{2}^{M}$.

Due to M>2, the relationship between $P_{b}$ and $P_{M}$ can be expressed as

(30) $$P_{b}=\frac{1}{k}\sum_{n=0}^{k}n\left(\begin{array}{c} k\\ n \end{array}\right)P_{M}=\frac{2^{k-1}}{2^{k}-1}P_{M},$$

where *n* is the length of the spreading code.

Therefore, SER $P_{b}$ can be expressed as

(31) $$P_{b}=\frac{2^{k-1}}{2^{k}-1}P_{M}=\frac{2^{k-1}}{2^{k}-1}\left(M-1\right)Q\left[{\left(\frac{N_{0}}{E_{b}\log_{2}^{M}}+\frac{2(k-1)}{3NT_{c}^{3}}\right)}^{-1/2}\right],$$

where $E_{b}$ is the bit energy.

In AGIVNs, the UAV data link faces a lot of EMI. Due to space limitations, we only analyze the anti-interference performance of the UAV data link under common EMI combinations. When the UAV data link is combined with WGN and SWCF interference, the total interference power spectral density of the system is

(32) $$J_{all}=N_{0}+J_{S},$$

where $J_{S}$ is the power spectral density of SFCE interference.

The SER $P_{b-2}$ can be expressed as

(33) $$P_{b-2}=\frac{2^{k-1}}{2^{k}-1}\left(M-1\right)Q\left[{\left(\frac{N_{0}++J_{S}}{E_{b}\log_{2}^{M}}+\frac{2(k-1)}{3NT_{c}^{3}}\right)}^{-1/2}\right].$$

When the UAV data link is combined with WGN, SWCF, and pulse interference, the total interference power spectral density of the system is

(34) $$J_{all}=N_{0}+J_{S}+J_{P},$$

where $J_{P}$ is the power spectral density of pulse interference.

The SER $P_{b-3}$ is

(35) $$P_{b-3}=\rho \frac{2^{k-1}}{2^{k}-1}\left(M-1\right)Q\left[{\left(\frac{N_{0}+J_{P}/\rho +J_{S}}{E_{b}\log_{2}^{M}}+\frac{2(k-1)}{3NT_{c}^{3}}\right)}^{-1/2}\right].$$

From the above analysis, Mary-MCM scheme improves anti-interference performance. In the next section, we simulate Mary-MCM scheme and compare the performance with DSSS scheme to verify the superiority of Mary-MCM scheme.

## 5. Simulation Results

We used MATLAB to simulate the proposed anti-interference scheme and compared Mary-MCM to DSSS.

### 5.1. Simulation Results

In our simulation, we used 50 UAV sensor nodes and 600 vehicle sensor nodes to simulate the proposed anti-interference scheme. Each sensor node uses the IEEE 802.11p protocol for data transmission. The communication frequency band is 5.9 GHz, and the source node and the destination node are randomly chosen in the network. Both the UAV and the vehicle use the shortest path map based movement (SPMBM) to plan the path, and assume that each sensor node does not discard the data due to factors such as cache and energy.

Simulation condition settings are shown in Table 1, and the Monte Carlo method is adopted to analyze the results.

The simulated signal to interference ratio (SIR) is defined as follows

(36) SIR=10log10(S/J),

where *S* is the signal power and *J* is the total power of interference, which can be expressed as

(37) $$J=N+\sum J_{I},$$

where *N* is the noise power and $J_{I}$ is the interference power.

We use SER as a measure of the performance of the anti-interference algorithm and treat $\mathrm{SER}<10^{-5}$ as normal communication. SER is defined as follows

(38) $$\mathrm{SER}=\frac{N_{e}}{N}\times 100\%$$

where $N_{e}$ is the error code in transmission and $N_{a}$ is the total number code of transmissions.

In our simulation, we simulated the common interference, and the interference signal simulation parameter settings are shown in Table 2.

In general, the EMI that the UAV data link faces is not single. Therefore, common EMI was combined to explore the influence of different interference combinations on anti-interference schemes. Common EMI combinations are shown in Table 3.

### 5.2. Impact of Different EMI Combinations on Anti-Interference Schemes

**Figure 17:**
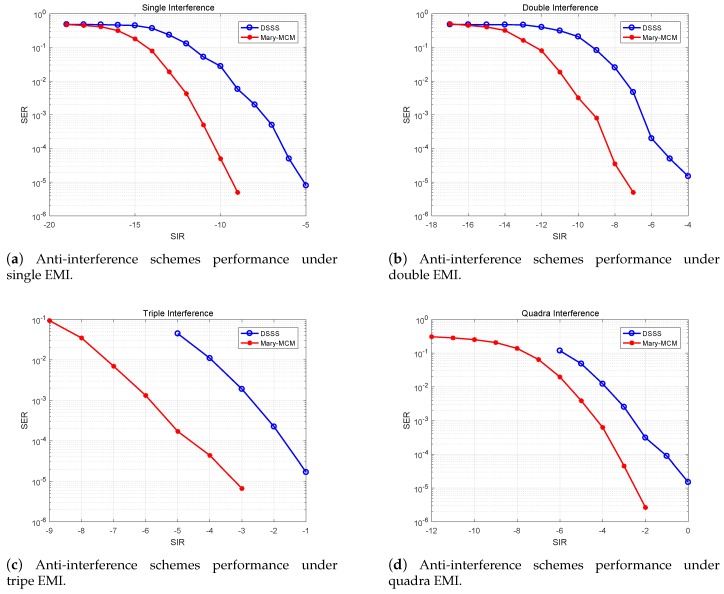
Anti-interference schemes performance under different EMI combinations.

Figure 17 indicates that the anti-interference performance of Mary-MCM scheme and DSSS scheme under different EMI combinations. From the data in Figure 17, it is apparent that with the increase of the amount of EMI, the performance of the two anti-interference schemes is decreasing. However, the performance of Mary-MCM scheme is always higher than DSSS scheme. Taking Figure 17d as an example, under the influence of quantum interference, the SIR of DSSS scheme can normally communicate is 0 dB ($\mathrm{SER}<10^{-5}$). However, the SIR of Mary-MCM scheme is only −2.6 dB. Therefore, Mary-MCM scheme improves the anti-interference performance of the UAV data link 2.6 dB.

In addition, as shown in Figure 17a–c, the SIR of Mary-MCM scheme for normal communication is −9.5, −7.5, and −3.3 dB, but the SIR of DSSS scheme for normal communication is 4.5, 3.5, and 2.3 dB. Thus, anti-interference performance has been improved by 4.5, 3.5, and 2.3 dB, respectively. With the decrease of the amount of EMI, the anti-interference performance of Mary-MCM is more significant. Therefore, compared with the traditional DSSS scheme, the proposed Mary-MCM scheme can transmit information more accurately in complex EMI environment.

### 5.3. Impact of Different Spreading Factors on Anti-Interference Schemes

**Figure 18:**
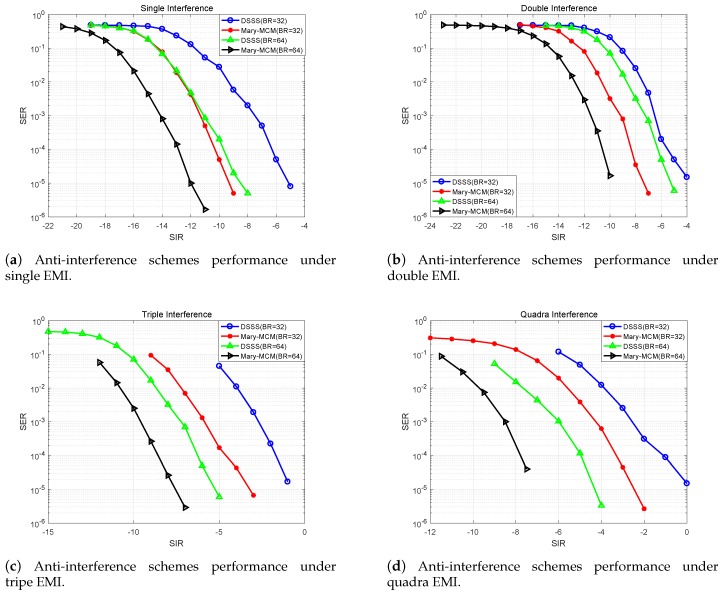
Anti-interference schemes performance under different spreading factors.

Figure 18 shows the impact of different spreading factors on anti-interference schemes. We extend the signal spectrum of Mary-MCM scheme and DSSS scheme by 32 and 64 times, respectively. As can be observed in Figure 18, we can extend the spectrum to improve the anti-interference performance of the UAV data link. With Figure 18d as an example, under quadra EMI combinations, the SIR that SRX can work normally is −7, −2.6, −4.5 dB, and 0 dB (Mary-MCM (BP = 64), Mary-MCM (BP = 32), DSSS (BP = 64), and DSSS (BP = 32), respectively). Therefore, the anti-interference performance of Mary-MCM (BP = 64) is 3.5 dB higher than that of Mary-MCM (BP = 32), and the anti-interference performance of DSSS (BP = 64) is 2.6 dB higher than that of DSSS (BP = 32). The anti-interference performance of Mary-MCM scheme is 34.62% better than DSSS scheme.

However, in AGIVNs, the spectrum resources are very limited, and it is impossible to expand the signal spectrum without limit. As showed in Figure 18a, under the WGN interference, the anti-interference performance of Mary-MCM (BP = 32) scheme and DSSS (BP = 64) scheme are similar. In addition, the anti-interference performance of Mary-MCM (BP = 32) is slightly better than DSSS (BP = 64), which is 1 dB higher. Therefore, the Mary-MCM scheme can effectively improve the spectrum utilization of the system, facilitate the frequency sharing of more services and accommodate more users.

### 5.4. Impact of Different Numbers on Anti-Interference Schemes

**Figure 19:**
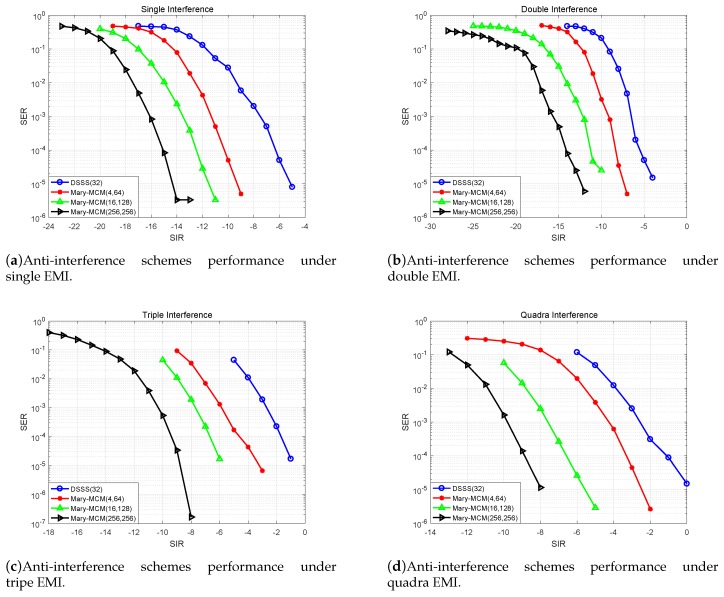
Anti-interference scheme performance under different numbers.

Figure 19 shows the impact of different numbers on anti-interference schemes. We extend both Mary-MCM scheme and DSSS scheme by 32 times. The Mary-MCM scheme is used to spread spectrum in 4-ary, 16-ary, and 256-ary. It can be observed in the figure that increasing numbers can improve the anti-interference ability of the UAV data link. This is because Mary-MCM scheme adopts M-ary spread spectrum technology to disperse the EMI power, so the signal interference power is only 1/*M* when the total EMI power remains unchanged.

In addition, as shown in Figure 19d, under the influence of quadra EMI, the SIR that can transmit information normally is −8, −5.6, −2.6, and 0 dB (Mary-MCM (4,64), Mary-MCM (16,128), Mary-MCM (16,128), DSSS). Therefore, the anti-interference performance of Mary-MCM scheme using 256-ary spread spectrum is 8 dB higher than that of DSSS scheme, and the anti-interference performance of the UAV data link is greatly improved.

The simulation results show that Mary-MCM scheme has better anti-interference performance than DSSS scheme. This is because the Mary-MCM scheme disperses the noise power by using M-ary spread spectrum, which reduces the system error rate, and enables the information to be transmitted more accurately. In addition, we use MIMO and MCM technologies in STX and SRX to improve the spectrum utilization and Mary-MCM scheme can better adapt to the multi-service, high-capacity, high-speed network environment.

## 6. Conclusions

In AGIVNs, the anti-interference technology of the UAV data link plays a critical role in reliable information transmission. In this paper, we design an UAV data link anti-interference scheme (Mary-MCM) based on the M-ary spread spectrum, the MCM, and the MIMO technologies to decrease the BER of the UAV information transmission. We use M-ary technology to expand the signal spectrum to improve the anti-interference ability of the UAV data link, and use MCM technology to improve the spectrum utilization while ensuring the anti-interference performance. In addition, we also adopt MIMO technology to improve the channel capacity. Simulation results show that Mary-MCM scheme has good anti-interference performance in complex EMI environment and can maintain the low BER under the condition of low SIR. Therefore, improving wireless sensor networks (WSN) performance by combining UAVs with the Internet of vehicles is an interesting research topic that deserves further study.

## Figures and Tables

**Table 1:** Mary-MCM scheme simulation parameters.

Category	Parameter	Value
Mary- MCM	Encoding	(2, 1, 7)
	Number of Carriers	4
	Subcarrier Spacing	1 MHz
	Modulation	BPSK

**Table 2:** Interference signal simulation parameter settings.

Interference	Parameter
WGN	SIR = [−25, 10] dB
RP	$\tau =5\times 10^{-8},T\;=\;5\tau$
NSR	SIR = [−15, 10] dB
SFCW	SIR = [−15, 10] dB

**Table 3:** Common EMI combinations.

Number of Interference	Type of Interference
Single Interference	WGN
Double Interference	WGN + RP
Triple Interference	WGN + NSR +RP
Quadra Interference	WGN + SFCW + RP + NSR
